# Recent Advances in Decellularized Matrix-Derived Materials for Bioink and 3D Bioprinting

**DOI:** 10.3390/gels9030195

**Published:** 2023-03-03

**Authors:** Huaying Liu, Yuxuan Gong, Kaihui Zhang, Shen Ke, Yue Wang, Jing Wang, Haibin Wang

**Affiliations:** 1 College of Life Sciences and Bioengineering, School of Physical Science and Engineering, Beijing Jiaotong University, Beijing 100091, China; 2 College of Life Sciences, Inner Mongolia University, Hohhot 010070, China; 3 National Institutes for Food and Drug Control, Beijing 102629, China; 4 State Key Laboratory of Toxicology and Medical Countermeasures, Beijing Institute of Pharmacology and Toxicology, Beijing 100850, China

**Keywords:** 3D bioprinting, dECM, dECM-derived bioink, tissue regeneration, regenerative medicine

## Abstract

As an emerging 3D printing technology, 3D bioprinting has shown great potential in tissue engineering and regenerative medicine. Decellularized extracellular matrices (dECM) have recently made significant research strides and have been used to create unique tissue-specific bioink that can mimic biomimetic microenvironments. Combining dECMs with 3D bioprinting may provide a new strategy to prepare biomimetic hydrogels for bioinks and hold the potential to construct tissue analogs in vitro, similar to native tissues. Currently, the dECM has been proven to be one of the fastest growing bioactive printing materials and plays an essential role in cell-based 3D bioprinting. This review introduces the methods of preparing and identifying dECMs and the characteristic requirements of bioink for use in 3D bioprinting. The most recent advances in dECM-derived bioactive printing materials are then thoroughly reviewed by examining their application in the bioprinting of different tissues, such as bone, cartilage, muscle, the heart, the nervous system, and other tissues. Finally, the potential of bioactive printing materials generated from dECM is discussed.

## 1. Introduction

Tissue engineering refers to research that uses bioactive materials combined with cells and cytokines to develop biofunctional materials for the repair and regeneration of damaged human tissues and organs and to achieve the restoration of tissue and organ structure and function. The decellularized extracellular matrix (dECM) has recently received much attention as a functional nature-derived material. It is a biological substance composed of extracellular matrix (ECM) proteins, cytokines, polysaccharides, and immunogenic components, such as cell fragments and nucleic acids, that can be removed from tissues and organs using decellularization techniques. The dECM has good biocompatibility and tissue origin specificity, which can promote cell survival, growth, proliferation, and provide a functional microenvironment. However, problems such as the insufficient mechanical properties of dECM and differences between batches of products will hinder its application and may influence the anticipated outcome.

Three-dimensional bioprinting can be defined as a technology that uses bioactive materials, such as biomaterials, cells and cytokines, assisted by computer technology [1], depositing bioinks to obtain 3D structures layer-by-layer after a long process of continuous modification [2,3]. Essentially, it is based on the expanded application of 3D printing. It can accurately deposit the specified cellular composition to a given site, reproduce the heterogeneity of tissues and organs, and even prepare density-flexible products via selective deposition to realize the composite of the mechanical microenvironment [4]. This technique allows us to prepare grafts that can be integrated into the damaged sites and bionic disease models [5], which can be applied to research on regeneration medicine and the pathogenesis of disease and drug screening in vitro [6]. The rapid advancement of this technology boosts personalized medicine and treatment; hence, 3D bioprinting is gradually emerging as an ideal approach in healthcare [7].

Producing bioink that contains bioactive ingredients, the material for 3D bioprinting, is crucial [8]. An adequate viscosity, high mechanical strength, biocompatibility, and biodegradability are typical characteristics of bioink [9]. Studies on bioink have been conducted, and a variety of materials, including both natural (collagen [10], fibrin [11], sodium alginate [12], chitosan [13], hyaluronic acid (HA) [14], and dECM [15]) and synthetic materials (polyethylene glycol (PEG) [16], polylactic acid (PLA) [17], and polycaprolactone (PCL)) [18], have been used in 3D bioprinting. One of the current optional materials utilized in 3D bioprinting, the dECM, has structural components and functional cytokines that can promote cell development [19]. Various dECM hydrogels produced from different tissues have been developed, which have been applied in the rebuilding of tumors [20], hearts [21], muscles [22], arteries [23], nerves [24], corneas [25], and bones [26]. Combining 3D bioprinting and dECM has shown tremendous potential and therapeutic effects in tissue repair and in vitro disease model construction. This review mainly introduces the latest advances in this area using dECM-derived bioink with 3D bioprinting. It lists the preparation and characterization process of dECM-derived bioink. Then, we briefly reviewed and summarized the existing 3D bioprinting technology. At last, the progress of this strategy in various fields is summarized, introspected, and prospected.

## 2. Preparation and Identification of dECM-Derived Bioinks

Extracellular proteins, polysaccharides, cytokines, and other substances form the ECM, a mesh structure dispersed around cells that provide the mechanical and biochemical cues essential for cell survival, growth, proliferation, and function. The dECM, a unique microenvironment, varies in each tissue and functions as a regulator of cellular life processes [27]. The biological structure and availability of its natural components provide the dECM with remarkable biocompatibility. It is ideal for 3D bioprinting, since it exhibits more significant bioactivity and reduced biotoxicity than synthetic materials. The extraction and identification of dECM from various tissues have been the subject of numerous studies [28].

### 2.1. Methods of Preparing the dECM

**Figure 1:**
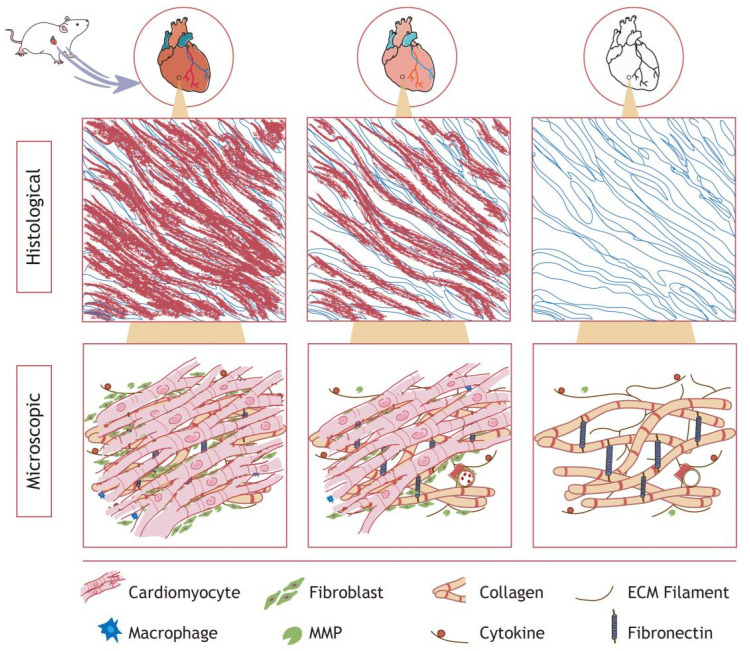
Schematic diagram of cardiac decellularization. Cardiomyocytes, epithelial cells, and blood cells are progressively removed, leaving a microenvironment rich in collagen, fibrous structures, and cytokines. The decellularization effect was approximately comparable in other organ tissues.

Decellularization aims to effectively remove immunogenic components from the tissue, minimize the damage to the scaffold, and maintain the integrity of the original structure [29]. Figure 1 illustrates the rat heart decellularization process, showing cells detaching from the ECM to obtain the dECM at the organ, tissue, and microscopic level. For any decellularization procedure, the ECM will suffer variable degrees of damage. The optimum protocol of decellularization differs among tissues due to their heterogeneity. In recent years, research on the decellularization of varied tissues has been carried out. At present, many methods and specific operating procedures for the preparation of dECM from various sources, such as cartilage, skin, brain and uteruses, have been explored, as shown in Table 1.

The physical method mechanically disrupts the cell membrane, lessens the usage of chemical solutions during decellularization, and mitigates tissue damage from solvent toxicity; other methods are needed to remove the leftovers. Physical methods, such as freeze–thaw, pressurization, and ultrasonic baths, are typically applied [52]. The freeze–thaw method utilizes intracellular ice crystals formed under low temperatures to destroy the cell membrane and dissolve the content at RT. Cryoprotectants are necessary to protect the ultrastructure of a cell. The pressurization method denatures and collapses the cell membrane by exerting pressure and is usually employed to promote the penetration and removal of detergents. Applying the ideal pressure level is crucial; low pressure cannot obtain the desired result, while high pressure could destroy the ECM structure and change its mechanical properties [53]. An ultrasonic bath can produce a high-strength shear force to cause internal particle disturbance collision and eventually break cells [36]. The superficial CO_2_/fluid technique can retain a significant quantity of GAG and collagen by using CO_2_ or fluids to lyse cells. Agitation produces mechanical forces that hasten cell detaching. In conclusion, using physical techniques alone rarely produces the best results, necessitating various methods to create high-quality dECMs.

Chemical methods disintegrate the cell membrane by destroying the chemical bond of proteins and lipids. Various reagents, such as hypertonic/hypotonic solutions, acid/base solutions, and ionic/nonionic/amphoteric surfactants, have been used in decellularization. Ionic surfactants that disrupt protein connections, such as sodium dodecyl sulfate (SDS) and sodium deoxycholate (SD), cause proteins to become distorted. Due to their performance, ionic surfactants are excellent at dissolving cells. However, the quantity of proteins and polysaccharides in the dECM is negatively affected, which could impact the stability of dECM scaffolds [54]. Non-ionic surfactants, such as TritonX-100 and Tween 80, are a class of mild decontaminants that work by severing lipid–protein interactions, which might confer less damage to the dECM and cell membrane than ionic surfactants. Therefore, they could effectively preserve a substantial portion of the protein content of dECM. The amphoteric surfactant 3-3-cholesterylaminopropyl dimethylamino-1-propane sulfonic acid (Chaps) is an efficient decellularization agent that may remove cells from fragile tissues and preserve the ultrastructure of the ECM. Both hypotonic and hypertonic solutions disrupt the cellular structure and exert a decellularizing effect by altering cellular osmolarity. Hypotonic solutions usually rupture the cell membrane, while hypertonic solutions separate cells from their surrounding matrix [55]. The enzymes often utilized in decellularization are nucleases, esterases, and proteases. Deoxyribonuclease and ribonuclease are the two most common nucleases. Compared to proteases, nucleases, and esterases can impart less damage to the protein framework of the ECM. Depending on the kind of tissue, it may be necessary to replenish the enzyme solution during decellularization, since cell leakage may limit enzymatic function. Moreover, the enzymes that remain on the dECM may cause adverse effects. Chelating agents bind to divalent cations (Ca^2+^ and Mg^2+^) to mediate ECM adhesion and interfere with proteins. Elution residues, including cell fragments and solutes of the reagent, need to be removed after treatment through other processes to prevent immune reactions. Presently, researchers prefer to use multiple strategies to obtain dECMs that resemble native tissues.

The dECM must also be sterilized to remove pathogenic components to prevent immune rejection in vivo and the alteration of the stability of in vitro investigations. Chemical perfusion/immersion (with ethanol, ethylene oxide, or peracetic acid) and physical approaches (dry heat, high-pressure steam, or γ-ray irradiation) are now commonly used sterilization methods. Since sterilization can change the characteristics of the dECM, it is also vital to use the right strategy to preserve the stability and biocompatibility of dECM components and structures [56]. High-temperature sterilization and the use of chemicals will inevitably denature proteins, while electron beam or gamma ray irradiation will damage the structure and mechanical properties of the dECM. Hence, it is still necessary to optimize the sterilization method to reduce the damage to the obtained dECM.

### 2.2. Methods of Characterizing the dECM

Identifying decellularization effectiveness is necessary to ensure product quality, primarily to check whether the cell debris has been removed and if the structure and components of the dECM are well-preserved. Hematoxylin–eosin staining can color the ECM components red and the nuclei blue. With the help of an optical microscope, one may determine whether nucleic acids are present on a histological level. Residual dsDNA could be directly linked to the remaining cells and trigger unfavorable host reactions. Quantitative assays have shown that less than 50 ng dsDNA per mg ECM dry weight is enough to achieve decellularization [57,58].

While achieving optimal cell removal is paramount, maintaining the structural stability and natural ingredients of the dECM is equally important. The loss of ECM components (such as collagen, cytokines, and HA) will affect the physical properties of materials and the specific microenvironment of cells. Many detection and characterization techniques have now been developed. Masson trichrome (MTC) is commonly used to identify the color of muscle fibers, collagen fibers, and nuclei. Collagen may be identified using Sirius Red staining, while glycosaminoglycans can be identified using Alcian Blue staining. Elastin can be determined using Verhoeff Van Gieson (VVG) staining, periodic acid Schiff (PAS) staining detects sugars in tissues, and Oil Red O staining can be used to detect lipids. Immunohistochemistry is also a commonly used detection method to reveal specific components [59]. Different methods used to characterize the dECM are essential primarily due to the unique components contained in tissues from different sources. Electron microscopy is also employed to assess the microstructure integrity of the dECM. The previously described electron microscopy methods include transmission electron microscopy (TEM) [60] and scanning electron microscopy (SEM). Furthermore, the protein and cytokine components can be thoroughly examined via proteomics, while the protein content may be precisely determined using Fourier infrared spectroscopy.

### 2.3. Preparation of dECM-Derived Bioink

Various dECMs, biomaterials, biomolecules, and cells are combined to create various bioinks. The processing steps for this material include choosing the right source, preparing the dECM, digesting, adjusting the concentration, and gelling when using the dECM as a raw material. Generally, tissues comparable to the application scenario are employed to lessen immunological rejection. The decellularization method used is as described previously, and the appropriate decellularization protocol was selected depending on the properties of the donor and host tissue.

Bioink forms crosslinked structures that contribute to the mechanical effects on cells, increasing mechanical strength and biological activity. Currently, crosslinking is accomplished via physical and chemical methods. Physical crosslinking is achieved using light and heat under specific conditions, while chemical crosslinking is achieved by adding chemical or natural crosslinking agents. Chemical crosslinkers may enhance the mechanical properties of tissues, but they are riskier to use than natural reagents. Examples of chemical crosslinkers include epoxy compounds, carbodiimide (CD), and glutaraldehyde (GA). They can impart detrimental effects on tissues and cells, resulting in severe immune rejection. Natural crosslinkers have slight effects on the dECM. Processing the dECM typically uses the following techniques to lessen the influence on its material properties. After being ground into a powder using liquid nitrogen, it is digested with a pepsin solution at the optimal pH (1.5–2.0), and the digestion process can be accelerated in a shaking table or under stirring. After digestion, the proper alkaline solution should be added to keep the pH neutral, and the mixture should be incubated at 37 °C to create a dECM hydrogel.

**Figure 2:**
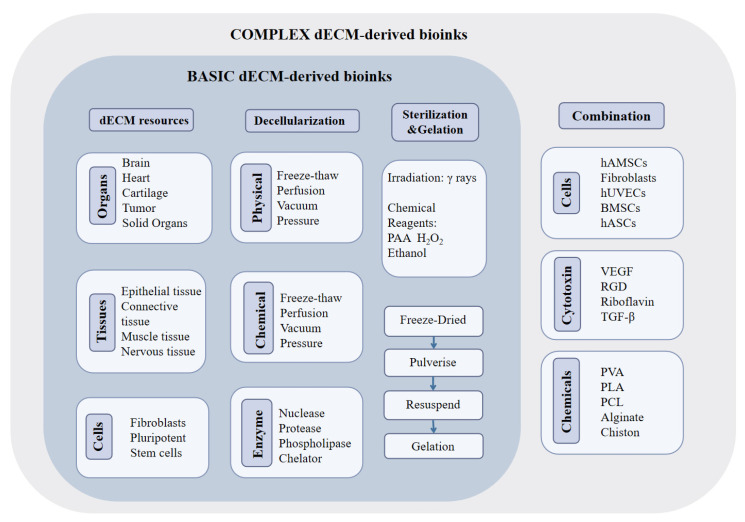
General procedure of preparation of dECM-derived bioink. Preparing complex dECM-derived bioinks requires binding cells, cytokines, and some chemicals to achieve better functionalization.

Selecting the appropriate bioink is crucial since the characteristics of the bioink used in the 3D bioprinting process dictate the biophysical properties of the printed porous or reticular structures produced. The dECM bioink may still include biochemical traces of the initial natural ECM [61]. Depending on the printing conditions, cells, growth factors, and various bioprinting materials are added to the bioink to help achieve better tissue repair and functionalization [62]. Generally speaking, there are basic and complex bioinks derived from dECMs. The dECM can also be obtained through a variety of optional decellularization operations in the cell culture environment. As mentioned above, after the preparation of dECM, basic bioinks can be obtained by sterilization and gelatinization. On this basis, some chemical components are compounded to adjust their physical properties, such as porosity and mechanical strength. Cells, peptides and cytokines are often added as bioactive ingredients, which helps to achieve the repair effect. The general process of creating dECM-derived bioinks is shown in Figure 2.

## 3. Properties of dECM-Derived Bioink

Due to the individualized nature and high precision of 3D bioprinting, there are specifications for the bioink that must be considered before it is used as a printing raw material. The dECM is a viable raw material for 3D bioprinting, since it is one of the sources of bioink. The richness of materials in a well-made dECM mimics the characteristics of native tissues, with a comparable microenvironment. However, specific requirements still need to be optimized for the dECM before 3D printing, such as its printability, biocompatibility, mechanical qualities, and degradability.

### 3.1. Printability

Printability refers to the ability of the bioink used to produce and maintain reproducible structures when 3D bioprinting technology is applied in the preparation of 3D scaffolds. Printability requires the printing carrier to have a certain mechanical strength, in which the cells can remain suspended and evenly distributed to form the bioink. The ability of quickly form and maintain a certain shape is necessary when printing [63]. Printability is essential for scaffold structures because it influences their mechanical strength and affects overall cell survival. The qualities of the bioink used, such as its biophysical properties and printability, heavily influence a material’s processability. Hence, printability is crucial in building precise 3D structural models. It is an important mission of bioink design to optimize and determine the parameters of the bioink with ideal printability [63].

Factors that affect printability include bioink properties, scaffold parameters, and the printing technology used [64]. The viscosity of the dECM bioink is determined by its tissue source, and the decellularization method affects its mechanical strength. The viscosity of the dECM-derived bioink can be controlled by adjusting the temperature and concentration. Previous studies have shown that the viscosity of a solution is positively correlated with its temperature, but it is impossible to achieve a consistent temperature during printing. The viscosity of the dECM bioink is also positively correlated with its concentration, but the correlation degree varies among different tissues. Adding materials with considerable mechanical strength is another tactic to modify dECM-related bioinks because the dECM does not have strong mechanical properties. Gelling is another step in the dECM bioink preparation process; choosing the proper crosslinking technique and agent is another method that can be used to manage mechanical strength. The deposition of bioinks and the mechanical characteristics of the scaffold might be influenced by the scaffold parameters, such as print orientation, pore size, and thickness. Bioink mixes, which change the component concentration to achieve optimal printing results, aid in improving printability [65].

### 3.2. Biocompatibility

Biocompatibility refers to the integration into the original position of the damaged part without triggering the immunological rejection of the implant, effectively interacting with its new microenvironment. The components of implantable biomaterials should not harm loaded cells, but instead promote cell adhesion, proliferation, cell-specific differentiation, and ECM formation for adequate tissue repair and regeneration. High cell activity must be ensured while loading cells for 3D printing to achieve greater bioactive effects and functionalization. In order to achieve high cell survival, bioinks with excellent shear rheological properties and suitable nozzle sizes are essential [66]. Due to the low biocompatibility of synthetic materials, a protracted inflammatory response at the implantation site may prevent the biomaterial from integrating with the host tissue. Hence, it is advisable to improve the biocompatibility of the materials using substances of non-biological origin [67]. Combining compounds with bioactive clues to suitable materials by coupling or deposition is a feasible approach. Biomaterials that match the mechanical properties of the injured tissue can also help with tissue restoration because they can translate mechanical cues into physiological responses [68].

For oxygen and nutrients to reach cells and keep them alive, the bioink structure should be reticular, associated with the integration and interaction of in situ damage to support healing and regeneration. A reticular structure can promote the development of loaded cells and reconstruct tissue-specific structures. Endothelialization and vascularization are also critical to prepare large-scale tissue analogues to maintain proper structures, functions, and integration [69]. Therefore, spatial structures constructed via 3D bioprinting-specific configurations can provide environmental clues to facilitate the expression of marker components and adequate functionalization.

### 3.3. Mechanical Stability

Bioinks have a rigid or elastic structure that, through the properties of the host tissue, can have good mechanical strength to protect loaded cells when printing and be stable enough to resist collapsing and other external forces after 3D printing before creating a scaffold. For 3D bioprinting, the mechanical characteristics of grafts should match the implant environment. Synthetic polymers and ECM components have been designed to realize the mechanical qualities of vascular structures to improve mechanical properties [70]. For bone tissue grafts, the pores with an appropriate size and interconnections should be distributed throughout the entire structure and have excellent mechanical strength to bear the force exerted by the surrounding hard and soft tissues. The behavior of a biomaterial under various situations is determined by its mechanical characteristics. Hence, tuning the mechanical properties of biomaterials affects the mechanical behavior of cells to obtain optimal tissue regeneration properties.

### 3.4. Biodegradability

Biodegradability refers to the ability of materials to decompose into simpler components under the effects of multiple molecules after exposure to the environment. Products from 3D bioprinting should gradually degrade as cells proliferate, migrate, and integrate with the ECM microenvironment. Therefore, the pace of degradation should correspond to the cell proliferation rate and establishment of a particular microenvironment, which parallels the new tissue synthesis rate. Dynamic ECM remodeling is a vital process caused by the interaction between grafts and tissues. Therefore, 3D-bioprinted products should guide the loaded cells’ survival, proliferation, and differentiation. At the same time, they should also progressively disintegrate and be replaced by new ECM produced by the cells. The dECM can offer a superior microenvironment for interacting with cells and the ECM. The rate of dECM hydrogel breakdown is also closely constrained; quick decomposition may produce unstable structural support, which cannot fully utilize the advantages of dECM bioink. In contrast, slow decomposition may obstruct cell diffusion.

Most dECM-derived bioinks may degrade into biological compounds, such as proteins, proteoglycans, and polysaccharides. Recent studies have shown that the dECM and its derivatives have outstanding biocompatibility and low toxicity, which support its use in biological applications. When synthetic materials degrade, the small molecule aggregation of degradation products could alter the pH and temperature of the local microenvironment. Rapid degradation may lead to changes in the microenvironment during cell adaptation. Cells and tissues can be harmed by immune responses brought about by the breakdown of synthetic and natural materials. The mechanical properties of hydrogels that are essential for cell migration and proliferation are related to their breakdown rate [71]. The signaling cascade of cell-biomaterial interactions is significantly influenced by the degradability qualities of biomaterials, which define the eventual healing effect of biological implants.

There are several types of 3D bioprinting technologies that are commonly used at present, including injection-based bioprinting, extrusion-based bioprinting, laser-based bioprinting and digital light processing (DLP). According to the characteristics of these types of technology, the parameters in the current application process are summarized in Table 2. Among the technologies, extrusion-based bioprinting is one of the most commonly used technologies for building high cell density and large tissues. For injection-based bioprinting, the resolution of this technology is relatively high, while it is only applicable to materials with low viscosity. In recent years, laser-based bioprinting is very popular because of its high resolution and cell viability, but its high cost limits its application. DLP has technical advantages, as is the case with laser-based bioprinting, but it still requires technical innovation due to its high cost and limited product size.

## 4. Application of dECM-Derived Bioinks in 3D Bioprinting

**Figure 3:**
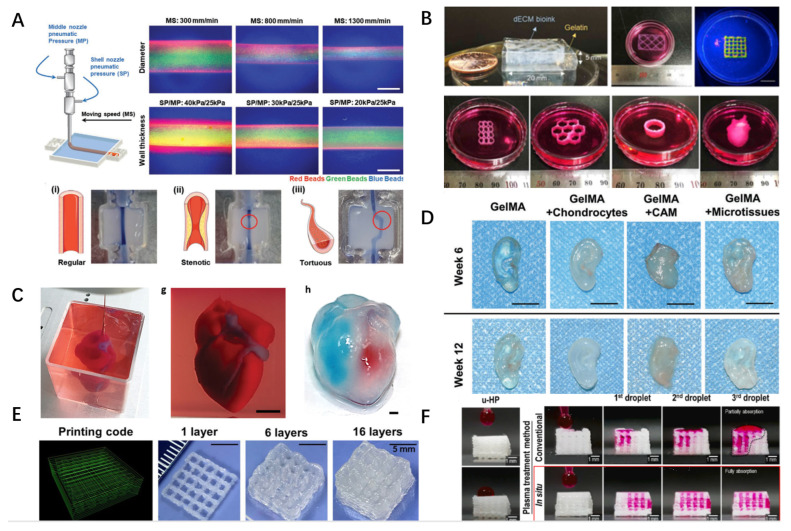
Examples of bioprinted products based on the dECM. (**A**) Based on the in-path coaxial printing technology, combined with vascular dECM (vdECM), controlled geometries are realized by controlling parameters, including (**i**) regular height, (**ii**) rigid, and (**iii**) torsional models (scale: 200 µm) (Reproduced with permission from Biomaterials; published by Wiley-VCH, 2019). (**B**) Using a granule-based printing reservoir in combination with skeletal muscle dECMs (mdECM) and vascular dECMs (vdECM) to produce a large volume of tissue constructs to achieve a printing chain, honeycomb, cylinder, and heart tissue (Reproduced with permission from Biomaterials; published by PERGAMON, 2019). (**C**) Cellularized hearts with a natural architecture were printed using human decellularized omental tissue (Reproduced with permission from Advanced Science; published by Wiley-VCH, 2019). (**D**) DLP bioprinting of auricular constructs was performed using a cartilage acellular matrix (CAM) and Gel-MA bioink combined with chondrocytes (Reproduced with permission from Advanced Healthcare Materials; published by Wiley-VCH, 2022). (**E**) Scaffolds prepared by kidney ECM-derived bioink (KdECMMA), which shows structural and functional characteristics of the native renal tissue (Reproduced with permission from International Journal of Biological Macromolecules; published by ELSEVIER BV, 2022). (**F**) The HA/PLLA/dECM composite scaffolds (ist-HP-e) showed promising mechanical and biological results (Reproduced with permission from International Journal of Biological Macromolecules; published by ELSEVIER BV, 2022).

Due to its capacity to mimic the physical properties of the tissue, 3D bioprinting has recently been used to construct in vitro disease models and tissue analogues. It is still a challenge to use the existing biomaterials to prepare biofunctional products. This highly depends on the selection of specific materials that can interact with active ingredients and the optimization of printing technology and parameters [84]. This section will provide explanations and examples of the uses of 3D bioprinting and discuss the developments of this technology in producing various tissues and organs. In recent years, 3D bioprinting products have included patches, scaffolds, organoids and organ-on-a-chip, which are used in the repair and reconstruction of various tissues and organs and the construction of disease in vitro models. The products and fields that have utilized 3D bioprinting in recent years are listed in Figure 3, and the parameters of 3D bioprinted-products mainly composed by dECM are shown in Table 3.

### 4.1. Heart

The heart is mainly comprised of myocardial tissue, heart valves, and blood vessels. The incidence of heart attacks and heart failure has risen in recent years; these diseases cause cardiac hypertrophy and local fibrosis, seriously impairing patients’ quality of life and posing long-term safety hazards. The considerable elasticity and strong mechanical strength of the heart mean that it is difficult for it to return to its original state under conventional therapy. At the same time, the demand for heart-specific drug testing and screening is increasing. Therefore, a tissue engineering strategy has been proposed [85]. Currently, in vitro 3D-bioprinted materials that mimic myocardial tissue are used in preoperative cardiac surgery simulations [86]. Traditionally, synthetic chemicals, polymers, and composites from natural sources have been used as material sources for tissue engineering. Due to the lack of similarity in composition and structure with biological tissues, achieving excellent repair and regeneration effects using these materials is challenging, and the biocompatibility of synthetic and natural materials with a single composition is poor. The integration of these materials with natural tissue damage is often insufficient, as they are often recognized as foreign bodies. For these reasons, we should promote the development and application of dECM for biological applications.

The dECM can also provide a non-restrictive microenvironment for cells, allowing them to engage in transport, migration, and reconstruct the ECM. Jang J et al. used the dECM from the left ventricular myocardium of porcine heart tissue to prepare bioink [87]. Compared with collagen type I (Col I), the mechanical strength of the dECM at the same concentration was lower (1.147–1.2 kPa). Using neonatal rat cardiomyocytes for 3D bioprinting, the ECM and Col groups showed no adverse effects. mRNA expression analysis showed that the expression of transcription factors (*Nkx-2.5* and *GATA4*) related to myocardial cell differentiation was enhanced in the group cultured in the dECM. The dynamic culture significantly promoted the expression of cTnT and Act2, which shows that the dECM highly represents the specific microenvironment of heart tissue. Moreover, dynamic culture conditions can enhance the maturation of cardiomyocytes.

Given that the dECM alone cannot achieve the elasticity and stiffness of the heart tissue, combining it with other materials is a strategy under investigation. Kim DH et al. combined heart-derived dECMs, laponite-XLG nanoclay, RPMI, PEG-DA, and a photo initiator, lithium phenyl-2,4,6-trimethylbenzoylphosphinate (LAP), to form a homogeneous hydrogel [88]. Rheological tests showed that the dECM bioink prepared with laponite had a higher modulus, was more viscous at rest and during flow, and showed extrudability, shape fidelity, and stack ability. Primary human cardiac fibroblasts (HCFs) and cardiomyocytes derived from human-induced pluripotent stem cells (hiPSC-CMs) were used to evaluate their cell viability in this scaffold. However, the scaffold constructed using this bioink has few biochemical clues and contains non-degradable PEG-DA.

In addition to providing mechanical strength, the electrical property of a scaffold is also important, providing electrical stimulation for cell pacing when needed. Through 3D bioprinting of dECM hydrogels mixed with electronic components, Tsui JH et al. prepared three kinds of bioinks to meet the electrical needs of the heart [89]. Among the bioinks produced, ECM-based hydrogels provided biological clues, and the mixture of liquid polydimethylsiloxane (PDMS) and graphite flakes provided electrical conductivity. The conductivity of the obtained hydrogel was 3–10 times higher than that of the natural myocardium, and it could promote cell functionalization and tissue maturation. Currently, using 3D bioprinting technology to manufacture myocardial chips is also an attractive project for high-throughput testing of drug sensitivity and resistance. Khademohosseini A et al. used 3D bioprinting as a type of technological support. Endothelial cells can be combined with micro-fluid perfusion bioreactors to build endothelial myocardial chip models, which can be applied in cardiovascular drug screening (Figure 3D) [90].

### 4.2. Blood Vessels

Most blood vessels are hollow tubular tissues composed of endothelial cells, smooth muscle, collagen fibers, and connective tissue and are responsible for transporting various substances, including oxygen. Vascular injury and dysfunction mainly include vascular rupture, occlusion, atherosclerosis, inflammation, and hemangioma caused by trauma, vascular sclerosis, and other diseases. The transportation of nutrition and oxygen is necessary for reconstructing large and complex tissues through 3D printing [23].

In recent years, polycaprolactone (PCL) [91], collagen [92], alginate [93], gelatin materials [94], poly-L-propylene-caprolactone (PLCL) [95], dECM derivatives, and growth factors [93] have become common materials for vascular regeneration. These materials are usually combined with human umbilical vein endothelial cells (HUVECs) [91] and human aortic smooth muscle cells (HASMCs). Modifying bioink with peptides and polysaccharides is an approach used to improve biocompatibility and functionalization [93]. Due to the compact structural characteristics of bone tissue, the research on its vascularization is ongoing, and some strategies have been proposed. Bertassoni LE et al. used Gel-MA to combine HUVECs and human mesenchymal stem cells (hMSCs) [96]. The hMSCs developed into pericytes, while the HUVECs demonstrated monolayer endothelial creation and vascular outgrowth based on a commercial CaP bioink, indicating their capability to produce functional and endothelialized whole-vessel bone scaffolds. Endothelial cell dECMs promote angiogenesis in the bone microenvironment. In vivo research revealed the importance of vascular infiltration and bone regeneration using a hybrid PCL/fibronectin/dECM scaffold (PFE) made from HUVEC-derived dECMs (HdECM), demonstrating that the environmental signals in the dECM could induce angiogenesis and osteogenesis [97].

The ECM plays an important role in angiogenesis. The protein and polysaccharide components in ECMs can regulate angiogenesis on a spatiotemporal scale [98]. Recently, Cho DW et al. utilized dECMs as a supporting bath for gel-embedded 3D bioprinting to construct tissue analogues (Figure 3C) [99]. Three-layer arterial tubular models of geometric shapes were successfully printed, including straight, narrow, and zigzag shapes, which contained HA and gelatin, and the fabricated products were stored in 3% VdECM bioink. The printed HUVECs, HCASMCs, and HDFs rapidly generated compartmentalized tissues within seven days, thus generating functional artery equivalents (AEs). The VdECM bioink promoted vascular cell activity, including cell proliferation, differentiation, and the local deposition of specific ECMs, allowing the constructed scaffold to respond to the biochemical and biomechanical stimuli that represent the physiological conditions of atherosclerosis. The design of the model could help us to directly detect the molecular signals related to the progress of local atherosclerosis. The presence of co-cultured HCASMCs and fibroblasts promotes endothelial dysfunction, monocyte recruitment, and the formation of foam cells. At the same time, a narrow and tortuous geometry can induce the deterioration of local atherosclerosis events and the formation of focal atherosclerosis lesions in turbulent areas.

### 4.3. Nerves

The nervous system is essential in regulating the overall function of the body, especially the central nervous system, which does not generally suffer from damage. However, nerve damage can also occur following trauma, exposure to neurotoxic substances, or cerebral hemorrhages. Moreover, the self-healing ability of nerves is limited. After a few nerves are damaged, other nerve cells in the system can compensate for this, but recovery to the original state is impossible. The large-scale repair of nerve injury is also challenging for tissue engineering. Recently, 3D bioprinting has shown its ability to treat and rebuild nerves. Materials such as PCL [100,101], chitosan, Gel-MA [102,103], gelatin, and PLCL [24] have been employed in the manufacturing of bioinks for 3D bioprinting to produce nerve guide conduits (NGCs) or patches with distinctive structures and mechanical strength. Reconstructing the nervous system requires good electrical conductivity. Valle J et al. produced a regenerating nerve cuff electrode (RnCE), utilizing poly (3,4-ethylenedioxythiophene): poly(styrene sulfonate) (PEDOT: PSS), the most used conducting polymer in bioelectronics combined with SU-8, an epoxy resin-based dielectric material, and silver precursor ink mixed with 10% glycerol. In rat models of chronic and acute nerve injury, the fabricated RnCE has been shown to heal sciatic nerve injuries. However, the unique regenerative PNI microelectrode sequence showed that the long-term implantation of this electrode could be harmful [104].

Cellularization or the attachment of bioactive cues could improve the biocompatibility and repair of a tissue scaffold. Anh D. Le et al. proved that using an MA hydrogel in combination with gingival mesenchymal stem cell (GMSCs)-derived NSSC/SCP-like cells (GiSCs) has a facilitative effect on neural function recovery and axonal regeneration [100,105]. Laporte LD et al. reported that bicyclic RGD peptides bound to PEG could act as biochemical cues and significantly promote fibroblast and nerve growth, directing a highly aligned growth condition [106]. Li XH et al. created a collagen/chitosan scaffold called 3D-CC-BDNF integrated with the brain-derived neurotrophic factor (BDNF); the scaffold contains a slow-release mechanism for treating spinal cord injury. The scaffold increased spinal cord regeneration at the lesion site and stimulated the regeneration of nerve fibers and synaptic connections [107].

A hierarchical structure more effectively simulates the nervous system compared to conventional homogeneous scaffolds composed of one kind of bioinks [108]. Shie MY et al. coated PCL conduits with PDA before submerging them in a dECM solution made from rat sciatic nerves at various concentrations to create nerve conduits. The dECM/PDA-coated PCL conduits had adequate mechanical strength, and could promote the upregulation of neuronal marker expression, enhance cell adhesion, proliferation, and differentiation, and even promote the regeneration of peripheral nerve defects [109]. Matsuda S et al. constructed scaffold-free nerve conduits developed from normal human dermal fibroblasts (NHDFs). The scaffold could promote the distribution of NHDFs in the nascent nerve and subsequent differentiation to functional Schwann cells (SCs) [110].

### 4.4. Muscles and Tendons

Muscles and tendons are two essential parts for sports; they are the major force-producing structures during a workout. Muscles are made up of muscle fibers that may contract, while tendons have a rigid linking structure made of thick connective tissue without the capacity to contract. A particular migratory region between soft tissue and bone is known as the tendon–bone interface (TBI). It can achieve self-repair following minor damage. When the injury volume is too large, auxiliary methods must be used for the interventional repair of muscles. Traditional muscular system treatments often involve sutures, autologous grafts, and allografts. After their repair, the injured tendon will exhibit adhesion and reduced strength and the problems of graft shortage and immune rejection will emerge. The current 3D bioprinting techniques have the potential to deliver functional, bionic-like structures to heal autologous bone injuries [111].

The uniaxial arrangement of muscle fibers works for the spatial distribution of myogenic cells and is essential for transporting nutrients and oxygen and maintaining muscle cell growth and alignment. Fabricating tissue scaffolds that resemble the skeletal muscle milieu with chemical substances is difficult. PLGA scaffolds can facilitate the proliferation and differentiation of C2C12 myogenic cells, promote myotube formation, and upregulate myogenic gene expression by simulating the ECM [112]. Microscale melt electrowriting (MEW), which can produce unique tissue morphologies and help direct cell alignment, has been used to write on the surface of a layered gold-coated nanofiber network. Graphene oxide is known to trigger myogenesis [113]. Park KD et al. used a graphene oxide hydroxyphenyl propionic acid euhedral gelatin hydrogel (GO/GHPA) as a bioink to print structures with an optimal microenvironment for the growth and differentiation of myoblasts [114]. GO can enhance the adsorption of fibronectin and albumin, upregulate intercellular signals, and induce spontaneous myogenic differentiation of C2C12 cells. Without differentiation factors, the GO/GHPA hydrogel increased the expression of muscle-specific proteins (MyoD and myogenin), proving its efficacy in promoting the myogenic development of C2C12 cells.

In addition, dECM-derived bioinks also showed enhanced biocompatibility, producing 3D-printed structures with good compatibility. They exhibit better functions in the regeneration of myotubes and new muscle fibers when combined with vascular-derived dECMs (vdECM) that contain HUVECs and skeletal muscle-derived mdECMs that contain human skeletal muscle myogenic cells (hSKMs) in a 1:1 ratio [115]. The scaffold offers a functional muscular structure that is highly active, contractile, and stiff. Meanwhile, alignment and delamination morphological cues could also be replicated by 3D bioprinting. Cho DW et al. implanted the scaffolds into a volumetric muscle loss (VML) rat model. They showed the formation of new muscle fibers, optimization of the damaged blood vessels and nerves, and 85% functional recovery in the VML. In their study, the 3D-bioprinted muscle structure showed improved function, which is reflected in the development of myotubes, improved cell survival, and the regeneration of muscle fibers. Kim GH et al. prepared a skeletal muscle-derived dECM-MA [116]. The results shows that the products displayed morphological and biological properties and could effectively arrange cells and the ECM, which simulates skeletal muscle tremor and leaching. The specific biochemical and morphological signals promoted C2C12 myogenic cells to differentiate into myotubes with highly consistent myogenic characteristics, which may lead to the effective functional regeneration of injured or damaged skeletal muscles. Skeletal muscle cell proliferation and myogenesis can also be increased by the dECM-MA, which could aid in developing new muscle fibers.

The traditional repair of tendon injuries often leads to a lack of nutrient infiltration and severe adhesion, which hinders the normal function of the tendon. Due to their density, achieving adequate nutrition infiltration in tendons and related TBI repair is challenging. Two scaffolds, one with a monolithic structure and another with a layered structure, were created by combining PLGA with a collagen/fibronectin hydrogel. They were found to be capable of sustaining the viability, proliferation, and tendonogenic differentiation of hADMSCs, with excellent TBI healing effects [117]. When combined with human primary adipose-derived stem cells (ASCs) in preparing bioink, nanofibrillar cellulose/alginate 3D bioprinting ASCs displayed tendon cell-like behavior [118]. Bovine Achilles tendon dECMs, or dtECMs, were also used to create a hydrogel [119]. The considerable collagen content of dtECMs results in their strong mechanical and biocompatible characteristics. To optimize printability, Zhao F et al. created a low-digestion state bioink with a high viscosity, utilizing porcine tendon dECMs and coupled them with rat bone marrow-derived stem cells (BMSCs), which exhibited high cell survival for an extended period [120]. Moreover, the team found in subsequent experiments that the proper digestion of tendon-derived dECMs could achieve superior cell spreading and proliferation.

### 4.5. Skin

The skin is rich in collagen and elastic fibers, with high elasticity and toughness. External injuries and plastic surgery result in a significant demand for artificial skin, which requires tissue-engineered skin substitutes [121]. Due to the intricacy of skin structures, 3D bioprinting, which has shown potential, still faces particular difficulties in artificial skin production [122]. Strontium silicate (SS) micropillars have been added to bioink for skin restoration, due to their capacity to induce angiogenesis [123]. Increased bioink stiffness stimulates myofibroblast activation and the expression of ECM-related proteins, while Gel-MA stiffness aids in promoting skin healing and prevents scarring (Figure 3A) [124]. Skin-derived dECMs are beneficial for skin restoration. Human dermal fibroblasts (HDFs), which may boost the expression of genes linked to skin shape and development in HDFs, were used by Park SA et al. to regenerate skin [125].

Porcine skin powder (PSP) may also be used to bioprint skin due to the skin-derived properties of bioink that contain PSP printing products. Lee SJ et al. prepared bioinks with a range of concentrations that contained alginate and PSP [126]. This bioink can induce cell functionalization and enhance collagen secretion. The skin shape and development-related gene expression were improved, and the encapsulated HDFs kept their morphological features and proliferated. Park JA et al. used fibroblasts connected to type I porcine dermal reticulocollagen hydrogels during 3D bioprinting and created a bilayer skin model by printing human keratin-forming cells onto fibroblast-mediated 3D protruding collagen microstructures [127].

### 4.6. Bone and Cartilage

#### 4.6.1. Bone

Bone tissue is composed of various cells and calcified intercellular substances, which can withstand great pressure and support the body. Bone injuries usually require surgical intervention, so grafts must have superior mechanical properties. The 3D bioprinting of artificial bone is a promising strategy for bone regeneration. The dECM may induce osteogenesis, enhance osteo-conductivity, and aid bone repair by fostering a conducive microenvironment. Although the mechanical properties of dECMs cannot directly induce bone formation, they may provide environmental signals that promote bone cell development [128]. Chemical materials are often used in synthetic multi-phase scaffolds for bone regeneration, such as PLGA/TCP/Mg (PTM) scaffolds with novel pores [129]. Dental stem cells (DSCs) have been shown to grow in printed scaffolds that contained 2% self-assembled octapeptide-conjugated magnesium phosphate AMP particles [130]. Bone cell growth has also been shown to be induced in GO/alginate/gelatin composite bioinks that contain hMSCs [131], gelatin/hyaluronic acid (HA)/hydroxyapatite (HAP) scaffolds [132], PCL/gelatin/nano-layered stents [133], and graded PLGA/nano-hydroxyapatite (n-HA)/gelatin (PHG) scaffolds [134].

Bioink with dECMs could induce cell-specific differentiation. Fan S et al. added 10% (*w*/*v*) polyethylene glycol diacrylate (PEGDA) and 0.25% (*w*/*v*) lithium acylphosphatephoto initiator (LAP) to pig tendon dECM hydrogels (tECM) to prepare bioink [135]. The PEGDA/tECM hydrogel produced had good hygroscopicity and appropriate mechanical properties. Compared with the control group, the proliferation ability of the cells grown on the PEGDA/tECM hydrogel increased with an increase in tECM concentration. The gel did not restrict cell migration, and the cell migration rate of the experimental group increased 3.6-fold compared to the control group. In the mineralization process, PEGDA/tECM hydrogels were found to be superior to PEGDA hydrogels in coordinating Ca and P_i_ deposition. In vitro, osteogenic differentiation experiments showed that osteogenesis-related genes were upregulated after 14 days of osteogenic induction. Eight weeks after the operation, the regenerated bone was successfully observed and analyzed using micro-CT scanning, and the maximum mineralized bone mass was found in the PEGDA/tECM group.

Kim GH et al. crushed porcine bone to demineralize, degrease and decellularize, and acidify it with methacrylic acid to enhance the mechanical strength of dECMs. They also prepared an alginate (Alg)/MA-dECM composite bioink [136]. The fabricated Alg/MA-dECM stent had a higher compression modulus (90.4 ± 14.9 kPa) than the control group (35.6 ± 8.9 kPa). The alginate component provides mechanical strength, while an increased dECM concentration can induce cell activity and functionalization. Compared with the control group, the 3D-printed structure increased the expression of the osteogenic genes *ALP*, *BMP-2*, cyanate, and *OPN*.

#### 4.6.2. Cartilage

Cartilage is a connective tissue with supporting functions and has strong toughness [137]. Simulation of cartilage surface morphology is a feasible strategy for modeling cartilage composition and mechanical strength (Figure 3B) [138,139]. Many studies have been conducted on bioink with additional dECMs for 3D bioprinting cartilage. Human adipose-derived stem cells (hASCs) and PCL have been bound together to produce PCL scaffolds covered with ECMs [140]. In addition, 2D nanosilicate (nSi) clay mixed with ECMs produced from pluripotent stem cells, known as “Nano-Engineered Ionic Covalent Entanglement Ink” (NICE), has also been studied [141].

Cartilage-derived dECMs are commonly used for cartilage tissue regeneration and repair. Isaeva EV et al. created a bioink that contains BMSCs, 4% collagen, and 2.5% dECM particles produced from articular cartilage [142]. Both cellular osteogenesis and chondrogenesis were induced by the human periosteal demineralized particles and dECM of cartilage origin. Additionally, some chemical materials are accessible when combined with bioink derived from cartilage dECMs. Based on aptamer hm69-mediated MSC-specific recruitment and growth factor-enhanced cytochondrogenesis, Yang Z, Zhao T, and colleagues produced a bifunctional 3D-bioprinted aptamer scaffold. This scaffold promoted cell adhesion and proliferation and attracted BMSCs more than other surfaces. Chemical modification of cartilage-derived dECM could also improve the properties of bioink [143].

### 4.7. Solid Organs

#### 4.7.1. Lungs

Lungs are challenging to duplicate in vitro due to their complex hierarchical structure and unique mechanical properties. With the outbreak of COVID-19, there is an urgent need for a bionic 3D model of the respiratory system [144,145]. Additional cells are required to improve bioink when 3D bioprinting a multilayered biological structure to replicate lung tissue [146,147]. To create 3D-bioprinted lung tissue scaffolds, Zhang Y et al. used silk fibers (SFs)and 2,2,6,6-tetramethylpiperidine-1-oxyl (TEMPO)-oxidized bacterial cellulose (OBC) nanofibrils made of bioink and crosslinked these with horseradish peroxide/H_2_O_2_ [148]. The OBCs directed the orientation of the lung epithelial stem cells, while the SF-OBC hydrogel scaffolds, in combination with chitosan and PCL, provided mechanical integrity and microenvironmental stability. Yamamoto T et al. developed a lung-airway model that included a flexible airway containing polyurethane foam with an iodinated contrast agent [149]. The motor platform demonstrated regular breathing patterns at various cycles. Losic D et al. developed a bioink with adjustable stiffness based on a porcine lung dECM hydrogel [150]. This bioink is qualified for the 3D cultivation of intrapulmonary BMSCs. These findings demonstrate that the 3D-bioprinted scaffold promotes cell–matrix interactions and that the lung hydrogel scaffold improved the adherence of cells in the culture.

#### 4.7.2. Liver

As the liver performs many functions in vivo, it is difficult to achieve the functionalization of 3D-bioprinted liver analogs in vitro. MA-Col I and thioacrylic acid (HA)-wrapped primary human hepatocytes and hepatic stellate cells were used to print 3D liver tissue mimics that were capable of exhibiting liver tissue functions with an appropriate response to APAP (acetaminophen) and maintaining urea and albumin production [151]. Moreover, bioink that contains 1% alginate, 3% cellulose nanocrystals (CNC), and 5% Gel-MA can print a honeycomb lattice and accurately deposit HepG2 hepatocellular carcinoma cell lines in a structure called a hepatic lobule mimic [152]. With the aid of silk fibronectin and a decellularized liver matrix (DCL), Sharma A and Rawal P created a 3D-printed scaffold comprising a silk gelatin DCL bioink (SG-DCL), which activates the Wnt/β-linked protein signaling pathway and creates a supportive environment for hepatocyte differentiation and function [153]. Furthermore, 3D liver microarrays have been reported to be a viable drug testing platform in vitro. Poly (ethylene/vinyl acetate) is the structural material primarily used to create 3D liver microarrays. HUVECs and differentiated human hepatocellular carcinoma cells (HepaRG) were encapsulated in liver dECMs and gelatin bioink, respectively. Lee H, Chae S, et al. printed a 3D liver microarray with various cell types for hepatocyte co-culture and the formation of vascular/biliary fluidic channels layer by layer [154]. The biliary fluid channel-equipped chip that they produced improved gene expression and function for the biliary system and the liver.

#### 4.7.3. Kidneys

The structure of the kidney is complex and includes the widespread distribution of blood vessels and nerve fibers; hence, 3D bioprinting of the kidney is challenging. Castilho M et al. fabricated tubular fiber scaffolds with small-diameter porous microstructures using MEW. HUVECs and conditionally immortal human proximal tubule epithelial cells (ciPTEC) were implanted into the tubule scaffolds, and cell self-assembly, specific ECM production, and renal function were tested. Their results showed that the kidney markers organic cationic transporter 2 (OCT2) and P-glycoprotein (P-gp) were expressed [155]. The repair and regeneration of renal tissue also extensively utilizes the dECM. The KdECM-MA bioink that comprised a renal dECM, gelatin, HA, and glycerol could replicate the kidney-specific microenvironment, and with the addition of particulate porcine kidney (pKECM) and human renal progenitor cells (hRPCs) can facilitate the functionalization of regenerated kidneys [156]. Kim BS et al. used the dECM from pig kidneys as the coating, hydrogel, and scaffold material, separately [157]. Compared to other materials, the coating significantly increased the expression of kidney-related genes. Moreover, the printed structures that served as hydrogels showed an internal cellular network topology and had dramatically increased kidney-related gene expression compared to HA hydrogels. As a scaffold, the hydrogel derived from pig kidneys demonstrated enhanced internal porosity, cell proliferation rates, and expression of kidney-related genes compared to Col I.

### 4.8. Tumors

Aside from organs, 3D bioprinting also enables the simulation of the natural tumor microenvironment, which could help us to improve the development of potential anticancer drugs and therapies. With high precision, 3D bioprinting can create heterogeneous tissues by depositing tumor-specific components [158]. Many strategies have been used to create in vitro target tumor models using 3D bioprinting. For example, MDA-MB-231 breast cancer cells were printed on a lipid-accumulating scaffold using HA-based bioink and human adipose-derived stromal cell (ASC) spheroids. Adipogenic genes were found to be highly expressed after 3D printing. This work illustrated the interaction between breast cancer cells and the ECM [159]. The team of Guo and colleagues created a tumor microenvironment that surrounds the tumor body with tumor-associated fibroblasts (CAFs) to overcome the low concentrations of Gel-MA and its limited printability using acoustic droplet printing [160]. The prepared bioink H4 and the modified H4-RGD ink exhibited favorable rheological properties. PDCs from 3D-printed non-small cell lung cancer patient xenografts grew fast and formed a tumor microenvironment in just seven days [161]. Chen H et al. prepared various PCL scaffolds. Colorectal cancer cells, CAFs, and tumor-associated endothelial cells (TECs) were seeded on 3D-printed scaffolds as a co-culture, forming the ECM to induce cell reprogramming. Normal stromal cells were activated and reprogrammed into tumor-associated stromal cells to construct a tumor microenvironment (TME) [162]. Bordoni M et al. prepared bioinks based on cellulose nanofibers (CNFs), alginates, and single-walled carbon nanotubes (SWCNTs). To construct the model, human neuroblastoma cells (SH-SY5Y cell line) were seeded on 3D-printed scaffolds. The scaffold provided electrical conductivity and promoted cell differentiation, causing the expression of *TUBB3* and *Nestin* genes [163].

Since it provides biochemical clues, the dECM has a significant advantage in constructing in vitro tumor models. To imitate the spread of kidney cancer to the liver, Wang SQ et al. implanted kidney cancer cells (Caki-1 cells) and hepatocytes (HepLL cells) in a mimetic liver microtissue made from a decellularized liver matrix (DLM)/Gel-MA scaffold [164]. The DLM retains the protein scaffold characteristics necessary for tumor growth and metastasis. The Young’s modulus of the DLM/Gel-MA stent was within the range of liver stiffness during liver cancer (5–60 kPa), and material exchange was realized in the microfluidic system to better simulate liver function and elucidate the progression of renal cell carcinoma metastasis to the liver. The results showed that the viability of cells cultured on the chip was more than 90%, while the HepLL cells continued to produce high levels of albumin and urea. The chip can also culture renal cancer cells to simulate the progress of cancer and then assess the therapeutic effect of various drugs. To create a tumor model, Chen Y and Xu L employed a bioink consisting of an adipose dECM mixed with Gel-MA and used it to grow MCF-7 cells [165]. The polypeptides and proteins in the adECM can carry cells, and the incorporation of the Gel-MA can improve the stability of hydrogels. The adECM/GelMA5050 scaffold also showed the same stiffness as malignant breast tissue (13 kPa). MCF-7 cells showed multicellular globular growth in the hydrogel, and their diameter increased to 60 µm on the seventh day. The increased expression of MMP-2 and MMP-9 in MCF-7 cells indicates that the malignant microenvironment of tumors in the 3D-bioprinted scaffold was enhanced. Compared with other groups, the expression of Ki67, a proliferation marker, in MCF-7 cells grown in the hydrogel scaffold showed a significant proliferation gradient similar to that of solid tumors. The growth rate of solid tumors in the 3D printing group was significantly higher and showed directional ECM formation. These results indicate that the 3D-printed MCF-7 tumor globules had improved tumorigenicity in vivo.

Researchers also developed a model that could mimic the mechanical strength of oral tumors. Yang S et al. used porcine kidney-derived dECM scaffolds to inoculate a human breast cancer cell line (MCF-7) and build a near-real breast cancer tumor model, which could simulate the tumor microenvironment in vivo. Their results showed that the expression of breast cancer- and hypoxia-related markers increased [166]. Moreover, concentric rings of tumor cells, vascular endothelial cells, and the acellular extracellular matrix maintained a radial oxygen gradient. These results showed that dECM-based scaffolds perform well in promoting growth and assisting angiogenesis.

Using 3D bioprinting to build an in vitro cancer-on-a-chip can combine the advantages of cost-effectiveness and microfluidic control to build a TME with complex biochemical clues, which can reduce the cost of tumor pathogenesis and drug screening [167]. For human glioblastoma (GBM), Cho DW et al. created a highly heterogeneous cancer analog chip (GBMs-on-chips) to test the response of patients to treatments [168]. The chip offers the following three clues of cancer pathological characteristics and microenvironments: biochemical clues provided by brain dECMs (BdECM), specific zoning structures of tumors, and biophysical clues provided by the oxygen gradient induced by the microvascular surroundings. The results showed that the abundance of biochemical clues of the BdECM in bioink affects the cancer cells. Moreover, compared with monolayer and spheroid culture systems, GBMs-on-chips are more effective in predicting the efficiency of test treatments and the systems with high bionic conditions when using drug combination treatment.

**Table 3:** The parameters of 3D-printed products.

dECM Source	Bioink Components	Key Properties	Technology and Parameter	Gelling Mechanism	Reference and Application
Heart	(1) cECM: cardiac extracellular matrix (2) Gel-MA	(1) hCPCs > 75% viability, (2) 30-fold increase in cardiogenic gene expression. (3) >2-fold increase in angiogenic	(1) Bioprinter (Envision TEC 3D-bioplotter Developer Series) (2) Pressure: 0.7–0.8 bar; Speed: 10 mm/s.	Adjust solution to pH 7.4.	[169] Personalized patch.
	(1) dhECM: decellularized human heart ECM (2) Gel-MA/ MeHA (Gel-MA -methacrylated hyaluronic acid)	Showed the potential of using GelMA– MeHA– dhECM (GME) and GelMA–dhECM (GE) hydrogels in mimicking post-MI cardiac tissue.	(1) A CELLINK Inkredible+ Bioprinter (2) a 22G nozzle; Speed: 75 mm/min; Pressure: 20–30 kPa.	Photocrosslinking (6.9 mW/cm2 UV irradiation) Then, mTGase solution for 30 min at 37 °C.	[170] Preparation of in vitro model.
Omenta	(1) Decellularized pig omenta, (2) Dielectric ink	(1) Built-in soft electronics (robustness up to 50%; elasticity below 20% strain). (2) The patch can withstand the expansion of the heart and operate properly.	(1) Extrusion-based bioprinter (2) The graphite ink: a 25 G conical needle at 5.5 kgf cm^−2^. The hydrogel-based ink and passivation ink: 27 G blunt needles at 1 kgf cm^−2^.	Adjust solution to pH 7.4.	[171] Provide electrical stimulation for pacing.
Skin	(1) Skin-derived acellular dermal matrix (2) Gel-MA	Provide more nutrients and a stiffer substrate, which facilitated cell activity, and might recruit host cells to accelerate neo-tissue ingrowth.	(1) Extrusion-based bioprinter (2) The printing speed was 5 mm/s and the pressure of the air compressor was set at 0.2 MPa.	Adjust the pH to 7.4; and add DMEM (10×) (volume ratio = 1:9) to adjust osmotic pressure.	[172] Injury repair and regeneration.
Kidney	(1) Kidney ECM- derived hydrogel methacrylate (KdECMMA) (2) Gelatin (3) Hyaluronic acid (HA) (4) Glycerol	(1) The sodium uptake capability of the human kidney cells was improved. (2) KdECMMA supports the formation of tubular and glomerular-like structures.	-	Photocrosslinking (add photoinitiator 2-hydroxy-1-(4-(hydroxy ethoxy) phenyl)-2-methyl-1propanone at the concentration of 0.5% to bioink solution).	[173] Bioengineer functional renal tissue construct for use.
Bone	(1) Decellularized cancellous bone (2) Tempo-oxidized cellulose nanofiber (TOCN) (3) Sodium alginate (SA)	Facilitates the development of cell proliferation, nutrient supply throughout the scaffolds and chondrogenic differentiation.	(1) 3D bioprinter (Rokit Invivo, South Korea) (2) Nozzle size is 24 gauge, moving speed is 5 mm/s.	Adjust solution to pH 7.4.	[174] Cartilage tissue regeneration
Meniscus	(1) Meniscus-derived dECM bioink	(1) Support for cell growth and fibrochondrogenic differentiation. (2) The mechanical and biological properties are similar to native meniscus.	(1) Stereolithography printing	Adjust solution to pH 7.4.	[175] Meniscus regeneration

## 5. Prospective

The ultimate goal of preparing biomaterials is to serve various clinical needs, so we need to produce safe and reliable ECM materials. There are still limitations in the applications of dECM. First, although many materials are available, the popularly used allogeneic- and xenogeneic-derived dECMs and chemical hydrogels are likely to lead to host immune rejection. In addition, it is necessary to understand the composition, properties, and structural characteristics of various materials from different tissue sources. Second, we need to select and optimize the decellularization method on this basis, such as using a more suitable nuclease or detergent and set up mild parameters of the physical method procedure to remove immunogenic substances and retain their structure. The residues of cell fragments, nucleic acids, and reagents, as well as the damage to the microstructure, will greatly affect the product properties. Finally, the sterilization method used will have an impact on the recipient tissues. Generally, tissues are sterilized using peracetic acid and ethanol or are irradiated after freeze-drying. At present, papers have summarized the sterilization methods that should be selected for dECM materials with specific properties to meet different application scenarios [176]. Although a detailed selection process can help us to obtain well-structured dECM scaffolds, these methods still damage the structure of the tissues and scaffolds to varying degrees and cause protein denaturation. Hence, better sterilization methods need to be explored.

The properties of 3D-bioprinted products can have a range of effects on tissue regeneration and recovery. As was already established, mechanical signals, the microenvironment, and surface morphology can all provide information about whether tissue development is being enhanced or hindered. However, it is unlikely that the mechanical properties of dECM materials will offer the majority of tissues the appropriate mechanical cues. Therefore, the dECM is commonly combined with chemical hydrogels to produce materials with improved mechanical strength. In addition to the mentioned issues above, bioink crosslinking also requires improvement. The most popular methods currently used, including chemical crosslinking agents, could inhibit cell proliferation and integration into tissues, which is unsuitable for tissue healing. The selection and long-term release of co-printed cells, cytokines, and chemical substances also need extensive investigation. At present, 3D printing technology is widely used, including injection-based, microextrusion-based and laser-assisted bioprinting, among which the microextrusion-based method is the most widely used in bioprinting. In application, it is often necessary to obtain the shear force caused by extrusion and the cell activity in bioinks.

The combination of 3D bioprinting technology and dECMs is a potential method for reconstructing large solid organs. Creating functionalized solid organs requires the accurate spatial placement of cells. Additionally, spatially specific environmental cues and mechanical strengths are necessary to improve tissue remodeling. The hierarchical design of the conduits for nutrition and oxygen transport, especially the architecture of the vascular network, is also crucial at the 3D printing level to imitate the complicated physiological structure within the organ of interest. As a result, the vascularized structure can carry nutrients and oxygen more effectively and provide assistance when the grafts integrate into the host injury milieu. The development of 3D printing and regenerative medicine will enable the implementation of this strategy in clinical settings.

At present, the 3D printing technology applied in biomedicine still faces some problems, such as the low cell survival rate, stringent requirements for the viscosity of bioink, and low resolution, among others. It requires the integration of multi-disciplinary knowledge to achieve accurate printing effects and obtain ideal structures. Moreover, for 3D bioprinting technology, reducing the technological impact on the active components of bioinks and increasing cell vitality in the printing process should also be taken into consideration.

## 6. Conclusions

With the development of regenerative medicine, dECM-based materials have shown potential applications in tissue engineering because they are taken from the original tissue and endowed with specific morphologic characteristics, adhesion sites, functional components, and signals, which promote tissue regeneration. In addition, 3D-bioprinted structures that contain dECMs can be used not only for transplantation, but also for drug screening, and pathology research. Therefore, the combination of dECMs and 3D bioprinting is a promising strategy for tissue and organ repair, organoid construction, disease model construction, and disease mechanism exploration.

## Figures and Tables

**Table 1:** Summary of latest studies on decellularization methods.

	Methods	Application	Process	References
Physical methods	Freeze–thaw	Human adipose tissue	Liquid nitrogen for 10 min, then 37 °C water bath for 30 min, 5 times.	[30]
		Porcine cartilage	Frozen and thawed repeatedly in liquid nitrogen for 6 cycles.	[31]
	Superficial CO_2_(ScCO_2_)/fluid	Porcine dermis	Tissue was pretreated and post-treated with superficial CO_2_ (ScCO_2_).	[32]
		Porcine nasal cartilage	Tissues were placed into a ScCO_2_ vessel system operated at 200–350 bar and 30–50 °C for 40 min.	[33]
	Ultrasonic wave	Porcine rib cartilage	Sonicated by water bath sonicator (Crest Ultrasonic) for 5 min.	[34]
		Human skin tissue	The ultrasonic bath was carried out at a frequency of 40 kHz, 2 h.	[35]
		Sheep osteochondral tissue	Ultrasonic bath (170 W, 42 kHz) and direct sonicator (80 W, 12 or 24 kHz) were used to treat some samples.	[36]
	Immersion and agitation	Canine uteruses from pregnant dogs	Immersion in decellularization solutions, then treated with agitator.	[37]
		Human adipose tissue	Tissue fragments were immersed in decellularization solutions, stirred at 37 °C.	[38]
	Perfusion	Porcine bile ducts	The process started with perfusion and recirculation of 1% SDS for 96 h, with changes every 24 h. Then, PBS was perfused for 24 h to remove remains of SDS.	[39]
		Rat pancreas	The protocol included 1% TritonX-100 for 60 min, 0.5% SDS for 120 min, 1% TritonX-100 for 120 min and 0.4 U/L DNase solution for 60 min. The flow rate was set to 5 mL/min.	[40]
	Vacuum	Canine infraspinatus tendon (IT)–humerus complex	Part of the tissue was rinsed with flowing PBS in a self-built VAS (0.1 mPa negative pressure) for 1 h.	[41]
	Pressure	Human dermal fibroblasts or collagen gels	Cell suspensions or biological tissues were subjected to high hydrostatic pressure (hHP) to reach a maximum hHP of 250 MPa and the hHP remained stable to control the compressive force.	[42]
Chemical methods	Ionic detergents	Mouse brain	Brains was soaked in a 10 mL solution of 1 % SDS, and stirred at 30 rpm for 24 h.	[43]
		Caprine ear cartilage	The tissues were placed in 4% Na-deoxycholate solution for 4 h at 37 °C with mild shaking, then incubated in 0.5% SDS solution for 24 h at 37 °C with agitation.	[44]
	Non-ionic detergents	Goat corneal tissue	TritonX-100 (0.5% in PBS) was perfused directly through the corneal tissue using a syringe pump unidirectionally at a constant flow rate of 50 µL min^−1^ for 48 h.	[45]
	Amphoteric detergent	Porcine and New Zealand rabbit corneas	Immersion of cornea in detergent solution (SDS, TritonX-100, Chaps), with solution/tissue ratio of 20:1(vol/weight), at RT and initiation of constant minimal agitation.	[46]
	Hypertonic/hypotonic solutions	Porcine kidneys	0.5 M NaCl solution (hypertonic solution) for 30 min, then 0.5% *w*/*w* SDS solution for 30 min, followed by deionized (DI) water (hypotonic solution) for 30 min.	[47]
	Acid–base/Alkaline and acid	Small intestinal submucosa (SIS)	Incubated in 100 mM of EDTA and 10 mM of NaOH (pH12) for 16 h, then incubated in 1 M HCl (pH1) and 1 M NaCl for 8 h.	[48]
Enzyme and chelators	Enzyme	Female pigs hemi-larynges	After incubated in a detergent solution (0.25% TritonX-100, 0.25% sodium deoxycholatein PBS), the tissue was washed and incubated with 2000 KU/L DNase and 0.1 g/L RNase at 37 °C for 24 h and the DNase/RNase step was repeated once more.	[49]
		Rat liver	4% TritonX-100, 3 h, CMF-PBS was added for 30 min, DNase and RNase solution circulated at 0.5 mL/min and 37 °C for 6 h.	[50]
	Chelators	The trabecular bone in the subchondral region of the arm of cows	0.1% EDTA (wt/vol) in PBS for 1 h and 10 mM Tris, 0.1% sodium dodecyl sulfate (SDS, wt/vol) for 6 h at RT.	[51]

**Table 2:** Overview of bioink parameters.

	Injection-Based Bioprinting	Extrusion-Based Bioprinting	Laser-Based Bioprinting	Digital Light Processing (DLP)
Particle diameter	10~50 μm	200~1000 μm	10~100μm	0~300 μm
Viscosity	3.5–12 mPa/s	1 × 10^6^~3 × 10^8^ mPa/s	1~300 mPa/s	10^3^~10^5^ mPa s
Cell density	<10^6^/mL	2~5 × 10^5^/mL	1 × 10^8^/mL	1 × 10^6^/mL
Storage modulus		10^3^~10^4^ Pa		10^3^~10^4^ Pa
Printing velocity	1 × 10^5^ droplets/s	10~7 × 10^5^ μm/s	200–2000 mm/s	1 mm^3^/s
References	[72,73,74,75]	[76,77,78]	[72,79,80,81]	[82,83]

## Data Availability

Not applicable.
